# Network pharmacology modeling identifies synergistic interaction of therapeutic and toxicological mechanisms for *Tripterygium hypoglaucum* Hutch

**DOI:** 10.1186/s12906-021-03210-8

**Published:** 2021-01-15

**Authors:** Dan Zhang, Yizhu Dong, Jintao Lv, Bing Zhang, Xiaomeng Zhang, Zhijian Lin

**Affiliations:** 1 Department of Clinical Chinese Pharmacy, School of Chinese Materia Medica, Beijing University of Chinese Medicine, No. 11 North Three-ring East Road, Chao Yang District, Beijing, 100102 China; 2 Center for Pharmacovigilance and Rational Use of Chinese Medicine, Beijing University of Chinese Medicine, Beijing, 102488 China

**Keywords:** Network pharmacology, Molecular docking, *Tripterygium hypoglaucum* Hutch, Mechanisms, Efficacy, Toxicity

## Abstract

**Background:**

*Tripterygium hypoglaucum* Hutch (THH) both has prominent efficacy and unwarranted toxicity in the treatment of autoimmune diseases. Nevertheless, its pharmacological and toxicological profiles still remain to be elucidated. In the current study, the network pharmacology approach was applied to identify synergistic interaction and mechanism of efficacy and toxicity for THH from a holistic perspective.

**Methods:**

The compounds from THH were collected using literature retrieval and relevant databases. After the production of putative therapeutic targets for dominant diseases and harmful targets of adverse reactions (ADRs) induced by THH, the protein-protein interactions (PPIs), topological analysis and pathway enrichment were established to distinguish the hub targets and pathways. Additionally, the binding activity of candidate ingredients with core targets were revealed by molecular docking simulation.

**Results:**

A total of eight bioactive components in THH were enrolled, and 633 targets were responsible for rheumatoid arthritis (RA), 1067 targets were corresponding to systemic lupus erythematosus (SLE), 1318 targets of ADRs were obtained. The results of enrichment analysis among THH-RA, THH-SLE and THH-ADR networks indicated that pathway in cancer, hepatitis B, rheumatoid arthritis, and PI3K-Akt signaling pathway might participate in THH for treating RA and SLE. Besides, the mechanism of ADRs that induced by THH were associated with viral carcinogenesis, p53 signaling pathway, PI3K-Akt signaling pathway, and so on. Whereas, these active ingredients of THH exerted the superior binding activities with crucial targets including STAT3, VEGFA, TP53 and MMP9 that functioned synergistically efficacy and toxicity as observed via molecular docking simulation.

**Conclusion:**

The present research preliminarily interpreted the synergistic interaction of therapeutic and toxicological mechanisms for THH through the comprehensive analysis of relationship and binding activity between primary components and core targets, providing a feasible and promising approach to facilitate the development of toxic and irreplaceable herbs.

## Background

As a typical Chinese herb with promising immunosuppressive and anti-inflammatory bioactivities, *Tripterygium hypoglaucum* (level) Hutch (THH), named as *Kunming-Shanhaitang* in Mandarin, is a traditional Chinese materia medica belonging to the family Celastraceae and the genus Tripterygium [1, 2]. According to the theory of traditional Chinese medicine (TCM), the medicinal effects of THH including dispelling wind and removing dampness, eliminating blood stasis and dredging collaterals, have been first dissertated in *Compendium of Materia Medica*, and it is warm in nature, acrid and bitter in flavor, with toxicity [3, 4]. Interestingly, with respect to morphologically similar species from Tripterygium genus, THH exhibits the similarity on therapeutic effects, clinical indications and toxicity with *Tripterygium wilfordii* Hook, besides, it has been widely affirmed in treating chronic autoimmune diseases due to mild pharmaceutical advantages and lasting immunosuppressive effects [5]. Currently, THH and its tablet preparations have played a desirable and beneficial role in the treatment of rheumatoid arthritis (RA), systemic lupus erythematous (SLE), and other autoimmune or inflammatory diseases for decades [6, 7]. The bioactive principles of THH, namely sesquiterpenoids, diterpenes, triterpenoids and so on, exert valuable and excellent pharmacological profiles of anti-inflammation, analgesia and immunomodulatory activities [8–10].

Although THH achieves considerably curative efficacy, with an overdose or prolonged exposure in clinic and its narrow therapeutic window, the application of THH has been impeded due to undesirable side effects and multiple organ toxicities including hepatotoxicity, nephrotoxicity, reproductive damage, and cardiotoxicity [11–13]. Accumulating evidence suggests that THH and its active ingredients could induce severe adverse reactions (ADRs), particularly hepatotoxicity and nephrotoxicity, these side effects can not only display the higher serum levels of alanine transaminase, aspartate transaminase, serum creatinine and urea nitrogen, but also are responsible for inhibiting CYP450 and the transporters [14–17]. Moreover, the clinical manifestation of reproductive system function impairments induced by correlative components of THH involving premature ovarian insufficiency, menstrual cycle irregularities, oligospermia and so forth [18–21]. Unfortunately, the gastrointestinal reactions including nausea, vomitus, abdominalgia and diarrhea, cardiovascular toxicities covering chest distress and palpitation had contributed to the limitation of its immense therapeutic potentials [22–24]. Remarkably, the irreplaceable merits of THH against rheumatic diseases are accompanied by side effects, therefore, its safety issues have raised major concerns. Nevertheless, current toxicological studies often focus on acute toxicity, reproductive toxicity and nephrotoxicity for its similar species from Tripterygium genus, *Tripterygium wilfordii* Hook, or some components from THH [25–29]. With this in mind, the approaches of network pharmacology combined with molecular docking were employed to distinguish bioactive components and predict key targets, for disclosing synergistic interaction of efficacious and toxic mechanisms for THH from a systemic and holistic perspective.

## Methods

**Fig. 1:**
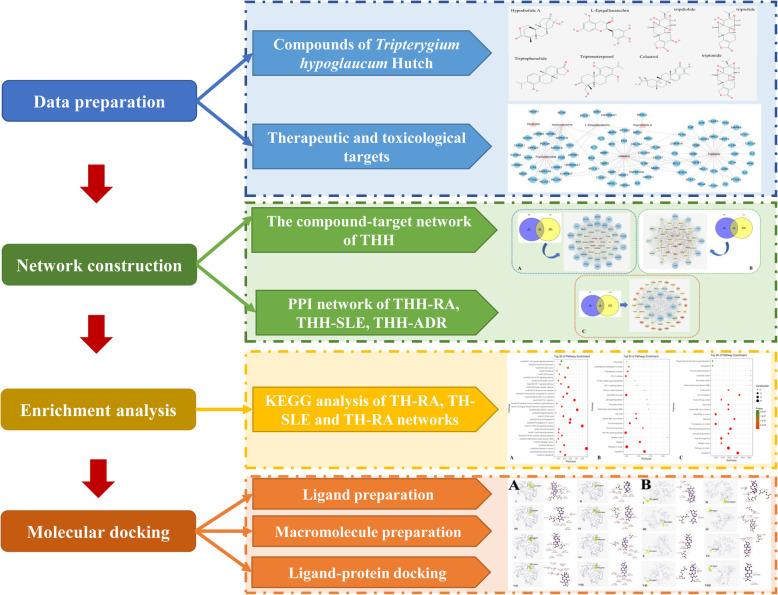
The flowchart of technical strategy in current study

The current study was proposed by four-step analysis including data preparation, network construction, enrichment analysis and molecular docking simulation, the flowchart of technical strategy in present study is illustrated in Fig. 1.

### Data preparation

#### Candidate components screening

As the world’s largest non-commercial TCM database available for drug screening in silico, herbal compounds of THH were collected and extracted from TCM Database@Taiwan (http://tcm.cmu.edu.tw) [30]. In addition, the potentially eligible compounds were recruited through the electronic databases of PubMed, the China National Knowledge Infrastructure Database, the Wan-fang Database, and the China Biology Medicine disc. Then, these components groups of THH were selected according to Lipinski’s Rule of Five, Veber’s rule and Muegge’s rule as potential therapeutic candidates [31, 32]. Moreover, the chemical information including MOL2 structure, canonical name and SMILES number were employed and downloaded for subsequent computational analysis from the PubChem Compound Database (http://pubchem.ncbi.nlm.nih.gov/) [33] and Traditional Chinese Medicine Systems Pharmacology Database (TCMSP, http://ibts.hkbu.edu.hk/LSP/tcmsp.php) [34].

#### Putative targets of THH

The targets data of main compounds from THH were identified and implemented from following databases: 1) Corresponding targets of all the promising components were captured from TCMSP Database. 2) The SMILES number of each candidate ingredient was imported into SWISS (http://www.swisstargetprediction.ch/) to acquire targets data of THH. This web server was designed to accurately identify potential targets of bioactive molecules according to a combination of 2D and 3D similarity measures with known ligands [35]. Targets with probability score ≥ 0.7 were selected for further analysis. 3) The predicted target profiles were also obtained from STITCH (Version 5.0, http://stitch.embl.de/) according to SMILES number of each molecule [36]. After discarding duplicates, the results of all targets prediction from above approaches were standardized into target genes names by utilizing Uniport sites (http://www.uniprot.org/) [37, 38], ultimately, these target genes from *Homo sapiens* were harvested altogether as targets database of THH.

#### Related targets of RA and SLE

According to Chinese Pharmacopoeia (2015 Edition) [39], the dominant diseases of THH were RA and SLE. Therefore, the keywords including “rheumatoid arthritis, rheumatoid, arthritis, rheumatoid nodule, rheumatoid arthritis” and “systemic lupus erythematosus, lupus erythematosus disseminates, Libman-Sacks disease, Libman Sacks disease, verrucous endocarditis” as the queries were searched for known therapeutic targets from the Online Mendelian Inheritance in Man (OMIM, http://omim.org/) [40], the Therapeutic Target Database (TTD, http://bidd.nus.edu.sg/group/cjttd/) [41] and Genetic Association Database (GAD, https://geneticassociationdb.nih.gov/) [42].

#### Associated targets of ADRs

Increasing experimental evidence has verified that THH could induce multiple organ toxicities including nephrotoxicity, hepatotoxicity, reproductive toxicity, cardiotoxicity and other side effects. The keywords of ADRs were searched in Comparative Toxicogenomics Database (CTD, http://ctdbase.org) to obtain the toxicological targets. Notably, CTD was a robust, publicly available research resource with over 30.5 million toxicogenomic relationships [43]. In addition, the searching entries of ADRs involved nephrotoxicity (“acute renal injury”), hepatotoxicity (“acute/chronic/drug-induced liver injury”), reproductive toxicity (“amenorrhea, menstrual disorder”), cardiac toxicity (“arrhythmia, palpitation”), hematological toxicity (“anemia, leukopenia”), gastrointestinal toxicity (“nausea, vomiting”), skin lesions (“rash, pruritus”) and so on.

### Network construction

First, the putative targets of active ingredients from THH were mapped to different targets’ group of dominant diseases and ADRs, which were regarded as potential efficacy and toxicity targets, respectively. Second, these candidate targets were inputted into the Search Tool for the Retrieval of Interacting Genes/Proteins (STRING, https://string-db.org/) to forecast three protein-protein interactions (PPIs), namely THH against RA (THH-RA), THH against SLE (THH-SLE) and THH caused ADR (THH-ADR), with the species limited to “*Homo sapiens*” and the confidence scores were higher than 0.4 [44]. Subsequently, the visualization and analysis of PPIs were undertaken through Cytoscape software [45]. Finally, the intersections of three PPIs including THH-RA, THH-SLE and THH-ADR were generated to identify core targets which were responsible for both clinical benefits and harms, and each hub target in the area of intersection was assessed and estimated with its typical central attributes: betweenness centrality (BC), closeness centrality (CC), and degree centrality (DC).

### Enrichment analysis

The Database for Annotation, Visualization, and Integrated Discovery Bioinformatics Resources (DAVID, https://david.ncifcrf.gov/, version 6.8) [46], a web-based online bioinformatics resource for the high-throughput functional annotation bioinformatics functional annotation and enrichment analysis, was conducted to perform for Kyoto Encyclopedia of Genes and Genomes (KEGG, http://www.kegg.jp/) pathway enrichment, with *p* value < 0.05 was considered statistically significant [47]. The visualization of enrichment analysis was illuminated through Omicshare cloud platform (http://www.omicshare.com/).

### Molecular docking simulation

Molecular docking simulation included three different steps, namely ligand preparation, macromolecule preparation and ligand-protein docking. The interactions and binding models between the candidate components and the crucial pharmacodynamic and toxic protein were verified by molecular docking via the Systems Dock Web Site (http://systemsdock.unit.oist.jp/) [48], which permitted molecular docking simulation for comprehensive characterization of ligand selectivity and action on a complex molecular network. First, the 3D molecular structure of each compound was captured from PubChem website and then imported to AutoDock Tools 1.5.6 (The Scripps Research Institute, Santiago, California and the United States) for removing water molecules, adding hydrogenate, and charging energy; the files of optimized small molecules were saved as mol2 format ultimately [49]. Second, the crystal structures and protein conformation of core targets were obtained and downloaded from Protein Data Bank (PDB) database (http://www.rcsb.org/pdb/home/home.do) [50]. Third, the definition of binding sites was established through an interactive molecular visualizer by clicking on the displayed structure model or residues listed. In addition, the docking scores which were reported by docK-IN ranged from 0 to 10 (from weak to strong binding), allowing a straightforward indication of binding strength. Briefly, the compounds with higher docking scores more than 7.0 pKd/pKi indicated that it possessed strong binding affinity with target receptors, and docking scores more than 5.0 pKd/pKi equaled moderate binding affinity [48].

## Results

### Identification of components and targets

**Fig. 2:**
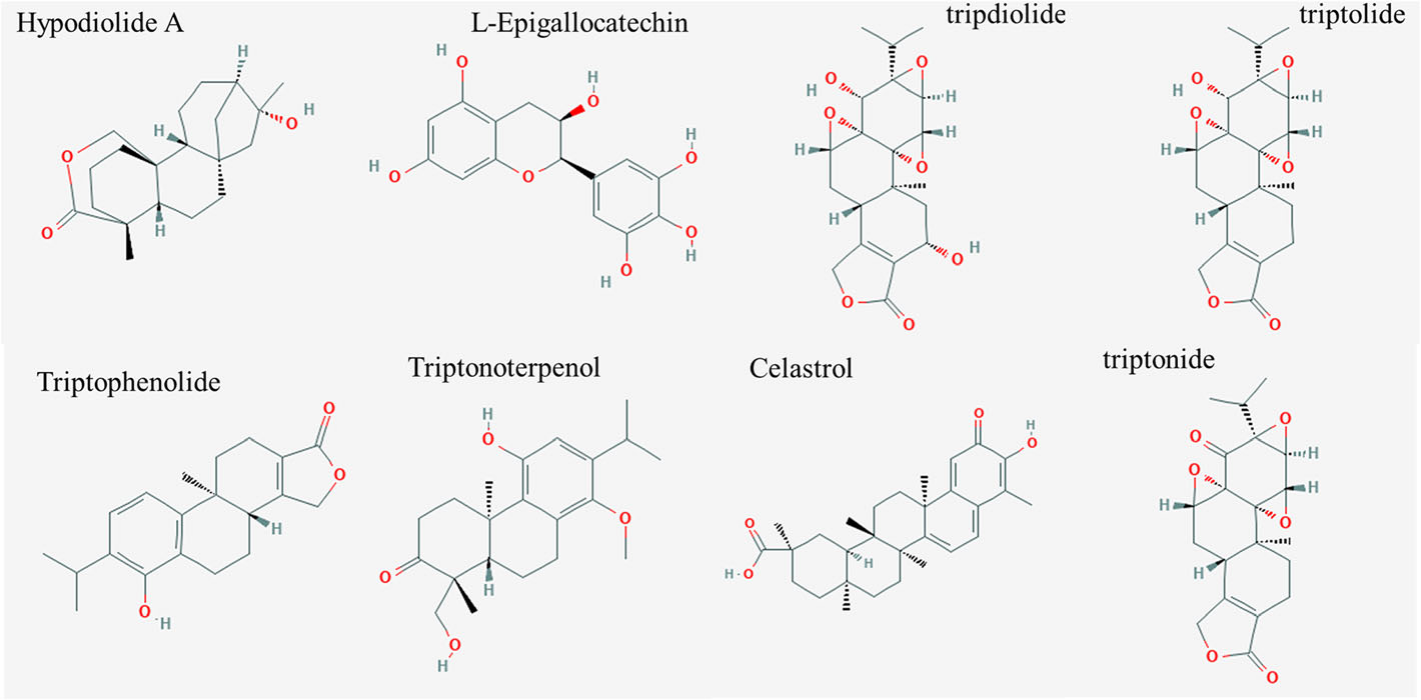
The chemical structure of active ingredients from THH

**Fig. 3:**
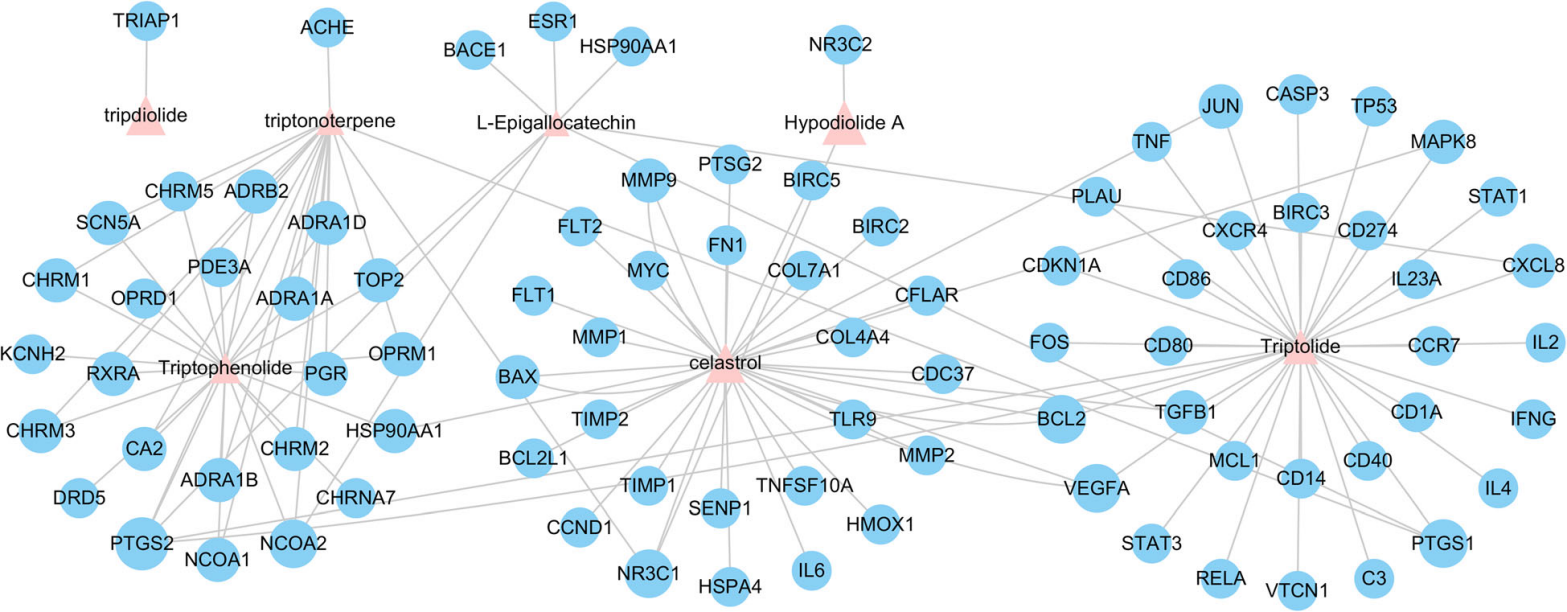
The compound-target network of THH. Note: The chemical constituents of THH are represented by pink triangles. Blue dots represent the direct target proteins of corresponding compounds

A total of 36 chemical ingredients of THH were obtained from the literature and TCM Database@Taiwan, ultimately, eight of them were regarded as bioactive ingredients by filtering with Lipinski’s Rule of Five, Veber’s rule and Muegge’s rule. The chemical information (chemical name, chemical formula, molecular weight and so on) and structure of active ingredients are displayed in Table 1 and Fig. 2, respectively. Altogether, 87 potential targets were acquired from previously described after removing the redundancy, and the compound-target network is depicted in Fig. 3.

**Table 1:** The chemical information of active ingredients from THH

Chemical name	Chemical formula	Molecular weight	ALogP	Num. H-bond acceptors	Num. H-bond donors	Num. rotatable bonds	Consensus Log Po/w	Log S (ESOL)	Log Kp (cm/s)	Bioavailability Score
Hypodiolide A (Tripterifordin)	C_20_H_30_O_3_	318.45	3.248	3	1	0	3.63	−4.23	−5.52	0.55
Hypolide (Triptophenolide)	C_20_H_24_O_3_	312.4028	4.63	2	1	1	3.91	−4.46	−5.33	0.55
L-Epigallocatechin	C_15_H_14_O_7_	306.267	1.779	0	0	1	0.42	−2.08	−8.17	0.55
Tripdiolide	C_20_H_24_O_7_	376.4	−0.23	0	0	1	0.90	−1.55	−9.23	0.55
Triptolide	C_20_H_24_O_6_	360.401	0.872	0	0	1	1.71	−2.15	−8.34	0.55
Triptonide	C_20_H_22_O_6_	358.385	1.259	0	0	1	1.93	−2.41	−8.02	0.55
Triptonoterpenol	C_21_H_30_O_4_	346.461	3.895	4	2	3	3.63	−4.59	−5.45	0.55
Celastrol (tripterine)	C_29_H_38_O_4_	450.619	5.9	4	2	1	5.12	−6.31	−4.83	0.85

### Network construction of dominant diseases and ADRs

**Fig. 4:**
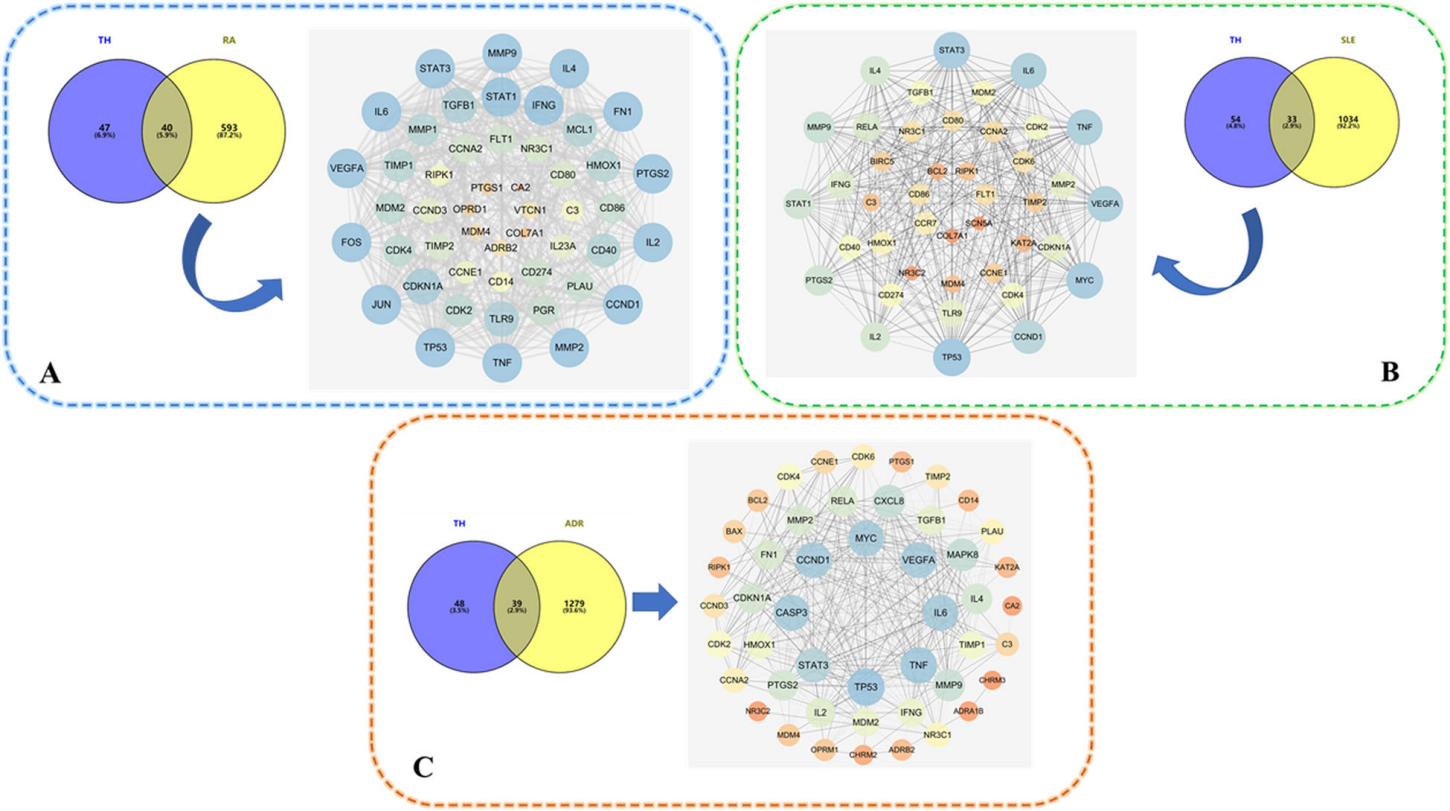
The PPI networks associating with the efficacy and toxicity of THH. Note: **a**: The PPI network of THH for treating rheumatoid arthritis (RA). **b**: The PPI network of THH for treating systemic lupus erythematosus (SLE). **c**: The PPI networks of THH induced adverse reactions (ADRs)

**Fig. 5:**
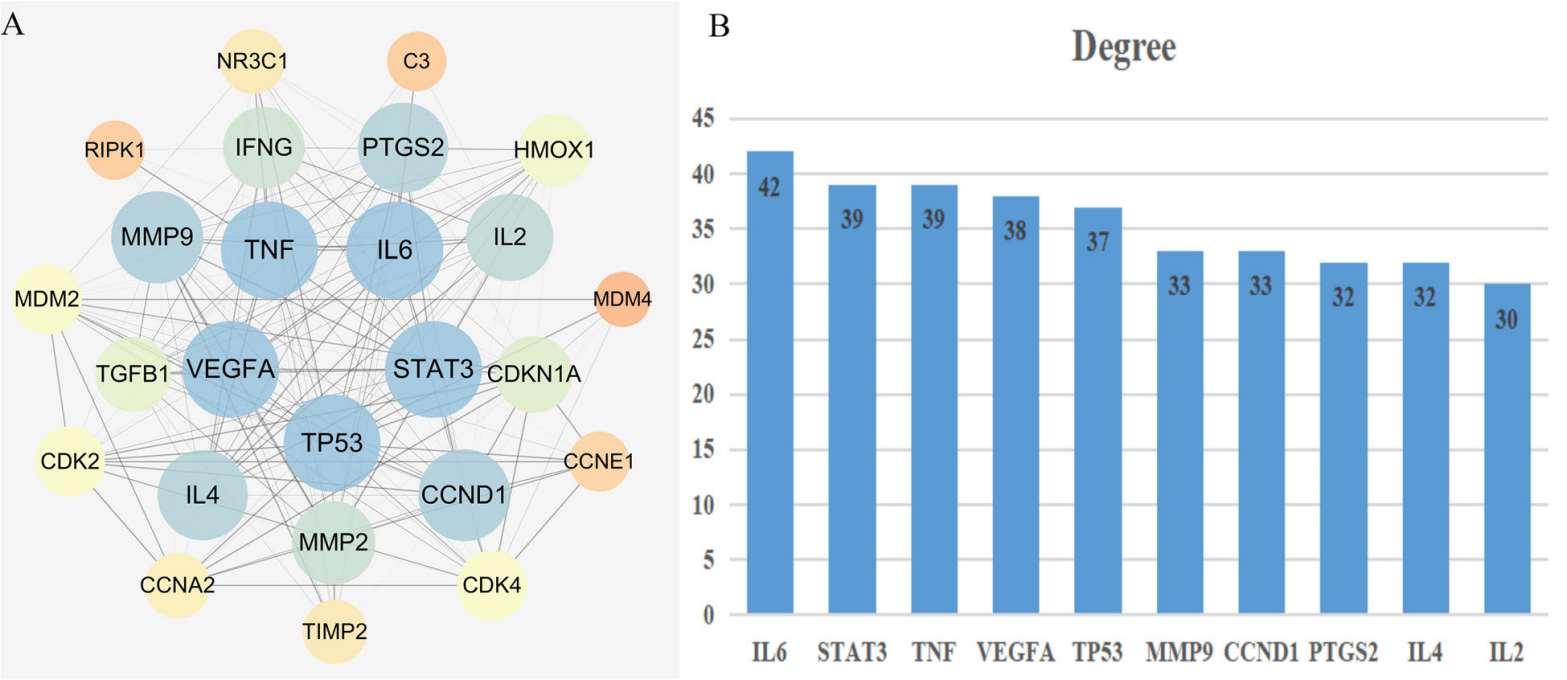
The PPI networks of RA-SLE-ADR for THH (**a**) and key targets with the higher degree (**b**)

The target prediction of THH in treating RA (targets number = 633) and SLE (targets number = 1067) and inducing ADRs (targets number = 1318) were achieved through online databases and relevant software. As illustrated in Fig. 4, the intersection of these putative therapeutic and toxicological targets of THH was generated and imported into STRING database to establish three PPIs including THH-RA, THH-SLE and THH-ADR. Based on these PPIs network, RA-SLE-ADR network was constructed for understanding the concurrent mechanism of efficacy and toxicity for THH. According to statistical analysis results of topological parameters, significant targets with the higher degree is summarized in Fig. 5 and Table 2. From yellow to blue, the degree of targets was increasing, and the thicker edges represented the stronger interactions, thereby indicating that the top mutual targets of THH had both beneficial and harmful functions at the molecular level.

**Table 2:** Topological parameters for key targets of RA-SLE-ADR (Top 10 by degree)

Name	Degree	Betweenness Centrality	Closeness Centrality
IL6	42	0.08073	0.8889
STAT3	39	0.05424	0.8421
TNF	39	0.05274	0.8421
VEGFA	38	0.03849	0.8276
TP53	37	0.03982	0.8136
MMP9	33	0.04720	0.7619
CCND1	33	0.02525	0.7619
PTGS2	32	0.01696	0.7500
IL4	32	0.02743	0.7500
IL2	30	0.02124	0.7273

### KEGG enrichment analysis

**Fig. 6:**
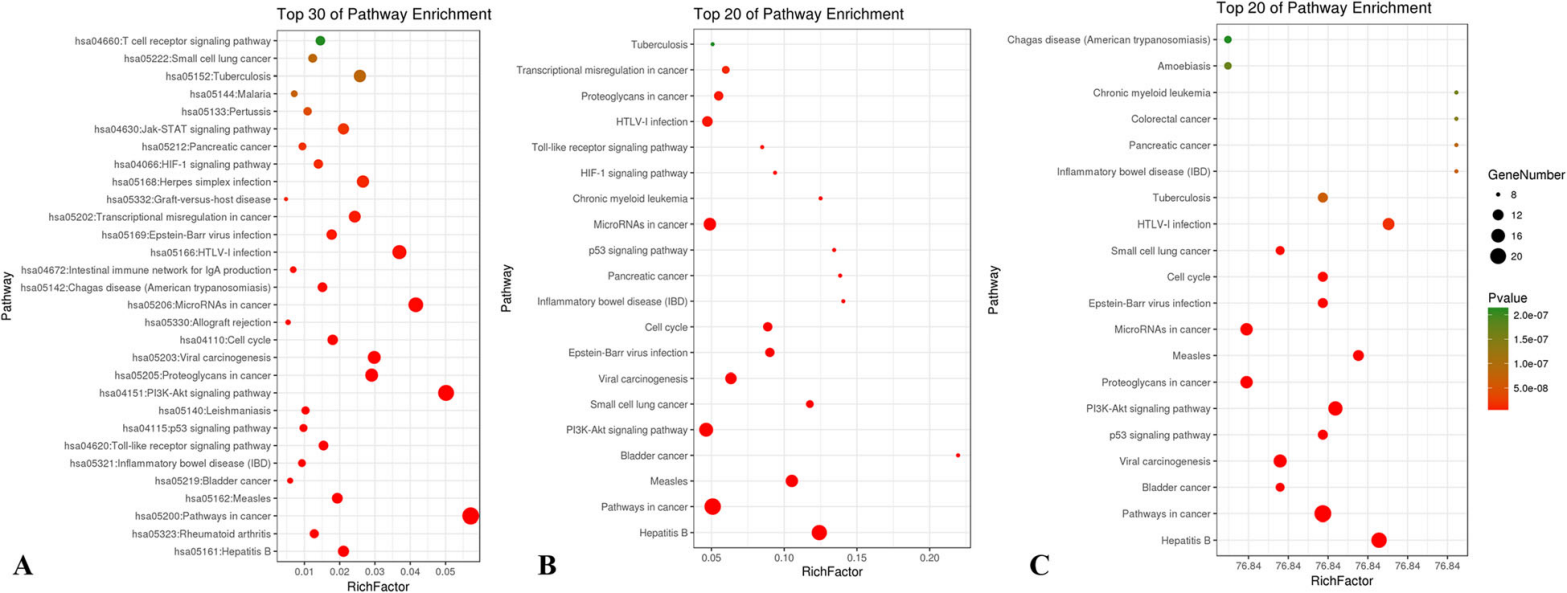
KEGG enrichment analysis of TH-RA (**a**), TH-SLE (**b**) and TH-ADRs (**c**) networks

The pathways enrichment analysis of candidate targets among THH-RA, THH-SLE and THH-ADR networks was performed by utilizing DAVID platform. The corresponding results elucidated that pathway in cancer, hepatitis B, rheumatoid arthritis, PI3K-Akt signaling pathway and measles were represented as pathways of THH for treating RA and SLE. Additionally, the ADRs caused by THH were associated with viral carcinogenesis, p53 signaling pathway, PI3K-Akt signaling pathway, pathways in cancer, and bladder cancer (Fig. 6).

### Molecular docking simulation

Among RA-SLE-ADR network, the key targets with higher degree including IL6 (Interleukin-6 receptor), STAT3 (Signal transducers and activators of transcription30, TNF (Tumor necrosis factor), VEGFA (Vascular endothelial growth factor), TP53 (Cellular tumor antigen p53), MMP9 (Matrix metalloprotein 9). Remarkably, in consideration of IL6 and TNF were inflammatory factors with the inferior specificity in current research. Consequently, STAT3, VEGFA, TP53 and MMP9 were identified as corresponding receptors that possessed both efficacy and toxicity simultaneously for further process of molecular docking simulation. The four targets were searched in PDB database, and their configurations were determined as 1BG1, 3DCY, 4DEQ and 1ITV.

**Fig. 7:**
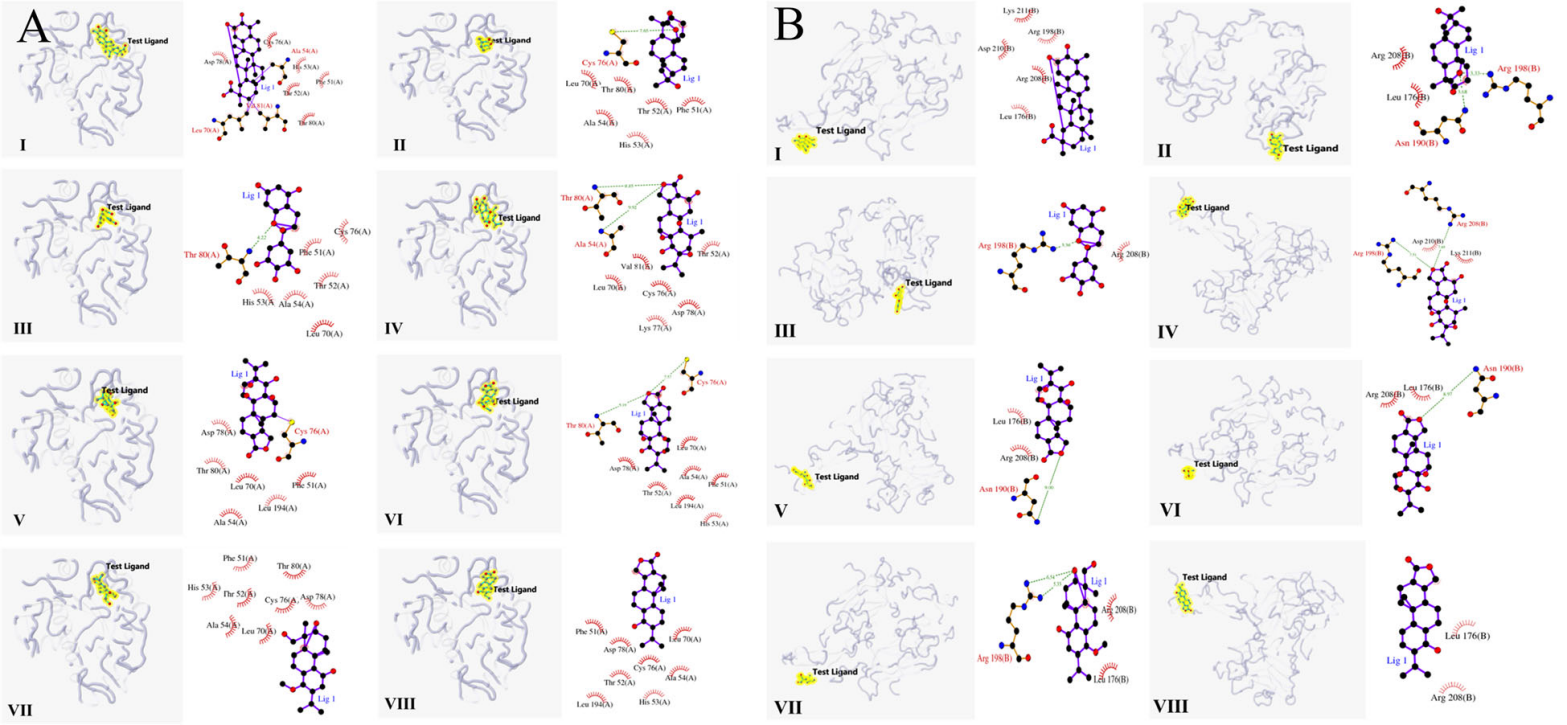
Molecular docking simulation between TP53 (**a**), VEGFA (**b**) and active principles of THH

To provide the deeper insight into the binding interactions between active principles of THH and these core targets, collectively, the results showed that each ingredient yielded moderate binding activity, suggesting there were the potentials of direct binding between these promising ingredients of THH and core targets. Especially, celastrol, L-epigallocatechin, triptonoterpenol and triptophenolide could achieve strong binding activity with MMP9 and STAT3. For example, the docking scores of core targets including MMP9, STAT3, TP53 and triptophenolide were 7.852, 7.869, and 6.152, respectively. Also, triptonoterpenol possessed the stronger binding capacity for MMP9, STAT3 and VEGFA, with the docking scores were 6.545, 6.567 and 6.152, respectively. The detailed of docking results is listed in Table 3. With regards to core targets in Fig. 7, TP53 and VEGFA well interacted with all active principles of THH. For instance, the binding mode of celastrol and TP53 was mainly reflected in two aspects, one was forming hydrogen bonding with Leu 70(A), Val 81(A), and Ala 54(A), the other was hydrophobic interactions with 6 amino acid residues, including Asp 78(A), Cys 76(A), His 53(A), Phe 51(A), Thr 52(A), and Thr 80(A).

**Table 3:** Docking scores (pKd/pKi) and relevant results of core targets and active principles of THH

No.	Entries	MMP9			STAT3				TP53			VEGEF	
		Docking scores	Num. H-bonds	Num. hydrophobic interactions	Docking scores	Num. H-bonds	Num. hydrophobic interactions	Docking scores	Num. H-bonds	Num. hydrophobic interactions	Docking scores	Num. H-bonds	Num. hydrophobic interactions
**I.**	**Celastrol**	6.238	1	1	6.514	0	1	5.634	3	6	**6.142**	0	5
**II.**	**Hypodiolide A**	4.803	0	0	4.583	1	1	5.741	2	6	**5.228**	2	2
**III.**	**L-Epigallocatechin**	**6.270**	0	0	**6.891**	0	2	5.329	1	6	5.040	1	1
**VI.**	**Tripdiolide**	5.398	1	0	5.072	0	2	5.746	2	6	5.466	2	2
**V.**	**Triptolide**	5.346	0	0	4.981	1	1	**5.985**	1	6	5.693	1	2
**VI.**	**Triptonide**	5.358	0	0	4.87	0	3	**5.965**	2	7	5.691	1	2
**VII.**	**Triptonoterpenol**	**6.545**	0	0	**6.567**	0	1	5.743	0	8	**6.152**	1	2
**VII.**	**Triptophenolide**	**7.852**	0	0	**7.869**	0	2	**6.152**	0	8	4.806	0	2

Note: The bold values were associated with the top three docking scores

## Discussion

THH, an anti-rheumatic herbal that has been widely used in China, is effective for treating autoimmune disease due to its excellent anti-inflammatory and immunosuppressive activities, however, the potential mechanisms of clinical efficacy and safety remain undefined and confused [50]. Therefore, we focused on the benefits and harms of THH during the treatment, at the molecular level, we were interested in the interaction and binding activities between hub targets and active principles of THH in the pathogenesis of dominant diseases and induced ADRs. On the one hand, eight kinds of medicinal chemistry from THH were selected as dominate chemical profiles, including hypodiolide A, hypolide, L-epigallocatechin, tripdiolide, triptolide, triptonide, triptonoterpenol, and celastrol. Moreover, the hub targets corresponding to these active ingredients were determined via combining network topological parameters with PPI network analysis. In total, four targets, namely, STAT3, VEGFA, TP53 and MMP9, might contribute to the pathogenesis of efficacy and safety. On the other hand, unexpectedly, these active ingredients of THH moderately bind to core targets to exert efficacy and induce toxicity simultaneously. Overall, therapeutic effects and herb-induced disease of THH were associated with the potential mechanisms that its active compounds played vital role in the regulation of STAT3, VEGFA, TP53 and MMP9. Herein, our study suggested that the combination of network pharmacology prediction and molecular docking simulation might provide some insights into the underlying mechanistic consequences and valuable approaches to characterize molecular mechanism of toxic herbs.

With regard to the active ingredients of THH which possess efficacy and toxicity concurrently, an anti-inflammatory and anti-oxidant activities is a bioactive compound, celastrol, is purified from herbs of the Celastraceae family, it can modulate the nuclear factor-kappa B (NF-κB), mitogen-activated protein kinases (MAPK), Janus kinase/ signal transducer and activator of transcription (JAK/STAT), Toll-like receptor (TLR) pathway and so on [51, 52]. In addition, the results of relevant study revealed that except for controlling of autoimmune inflammation, celastrol has the potential of pharmacological treatment of obesity as leptin sensitizer in silico drug screening methods [53, 54]. By contrast, celastrol that involves in inhibition of hERG channel activity and apoptosis could induce cardiotoxicity via the techniques of patch clamp, integrated metabolomics and network toxicology [55, 56]. Besides, celastrol and triptolide have toxic potencies on hepatocytes that mediated by hepatic CYP450s, affect embryonic development of zebrafish in μM concentrations [57, 58]. Similarly, triptolide and tripdiolide participate in the production of cytokine and chemokine to significantly ameliorate lupus nephritis in (NZB x NZW) F1 mice, triptolide and triptonide can upregulate the expression of IL-37 via the activation of the ERK1/2 and p38 MAPK pathways to achieve anti-inflammatory functions [59, 60].

From the perspective of core targets, strikingly, as a predominant member of extracellular polypeptide ligands like interleukin-(IL)-6-family, STAT3 protein is central in determining the inflammation and immunity, fulfilling fundamental functions in immune cells compared with the other members [61–63]. Additionally, overwhelming evidence suggests that STAT3 has the largest spectrum of potential activators, including various cytokines, hormones, and growth factors. For instance, it has also been implicated in many cellular processes including the transcription or opening of mitochondrial permeability transition pore, cardiomyocyte function, cardiac microenvironment, communicates in cardiomyocytes, cardiac fibroblasts, endothelial cells, smooth muscle cells, inflammatory cells and cardiac neurons [64, 65]. Other peculiar characteristics of STAT3 cover its ability in inactivation of embryonic lethality, reduction of tubulointerstitial and renal lesions, regulation liver progenitor, development of lung fibrosis [66–69]. Consequently, these functional properties of STAT3 is relevant to unwarranted side effects of THH. Subsequently, VEGFA may be promising markers of RA with clinical significance because it is the most potent proangiogenic molecule promoting the angiogenic phenotype, and it has been proven that its polymorphism is associated with RA susceptibility among Chinese and Mexican patients [70–73]. Also, it has been demonstrated that VEGFA implicated in regulation of cardiovascular development, male fertility, hepatic angiogenesis, glomerular filtration barrier through researches in the field of pharmacology, gene polymorphisms and clinical data [74–79]. TP53 (Cellular tumor antigen p53), a stress response gene, is involved in diverse cell death pathways and its activation, and it always correlates with rheumatoid arthritis, neuronal damage, acute myeloid leukemia, hepatocellular apoptosis [80–84]. As a member of proteases family that is secreted as inactive zymogens, it has been widely accepted that Matrix metalloproteinase 9 (MMP9) is critical in regulating extracellular matrix during the degradation of various extracellular matrix proteins, and it is possible pathogenic factor and potential therapeutic target for multi-system disorder [85–87].

Recently, network pharmacology has made a tremendous advance to drug discovery and TCM development [88, 89]. Coincidentally, it is consistent with unique characteristic of TCM or formulas that highlights holistic therapy and synergistic effect via multiple targets and pathways. And this approach has been applied in drug development in consideration of both efficacy and toxicity. Given this, network pharmacology holds the promise of expanding current opportunity space for druggable targets, and deciphering potential mechanism based on the prediction of the target profiles and pharmacological actions of herbal compounds [90–92]. Remarkably, molecular docking simulation is an important method in structural molecular biology and computer-assisted drug design [93]. Herein, the combination of these techniques can not only provide powerful and feasible means for investigating the mechanisms for bioactive ingredients, but also reveal the relationships, interconnections and binding free energy between chemicals and corresponding targets [94–96]. According to the results of the molecular docking and protein-ligand interaction profiles in present research, among the four core proteins, TP53 and VEGFA well interacted with all active principles of THH via more hydrogen bonds and amino acid residues.

## Conclusion

In conclusion, the integrated strategy of network pharmacology and molecular docking simulation was used to illustrate active ingredients and molecular mechanisms of clinical efficacy and safety for THH. Accordingly, therapeutic effects and herb-induced disease of THH were associated with its active compounds, which had the superior binding activities for regulating STAT3, VEGFA, TP53 and MMP9. Taken together, current research provided a comprehensive overview for toxic herbs whose benefits and harms should be balanced during clinical treatment. However, these findings of our results are warranted to validate by further pharmacokinetics and pharmacological researches.

## Data Availability

Specific study data are available from the authors on request.

## Abbreviations

ADRs: adverse reactions
BC: betweenness centrality
CC: closeness centrality
CTD: Comparative Toxicogenomics Database
DAVID: The Database for Annotation, Visualization, and Integrated Discovery
DC: degree centrality
GAD: Genetic Association Database
IL6: Interleukin-6 receptor
KEGG: Kyoto Encyclopedia of Genes and Genomes
MMP9: matrix metalloprotein 9
OMIM: Online Mendelian Inheritance in Man
PDB: Protein Data Bank database
PPIs: protein-protein interactions
RA: rheumatoid arthritis
SLE: systemic lupus erythematosus
STAT3: Signal transducers and activators of transcription 3
STRING: Search Tool for the Retrieval of Interacting Genes/Proteins
TCM: traditional Chinese medicine
TCMSP: Traditional Chinese Medicine Systems Pharmacology Database
THH: Tripterygium hypoglaucum Hutch
TNF: Tumor Necrosis Factor
TP53: Cellular tumor antigen p53
TTD: Therapeutic Target Database
VEGFA: vascular endothelial growth factor
